# Revivification of Microscopic Animalculæ

**Published:** 1843-01

**Authors:** 


*Revivification of Microscopic Animalculæ.*—Milne Edwards read a report on a memoir of Doyere on this subject.

It is well known that when a few drops of water are sprinkled on moss which has been kept dry for months or years, certain microscopic animalculæ make their appearance; but it has not been decided whether these little animals are really brought to life again, or whether their ova had not remained concealed in the moss, and were afterwards vivified.

Doyere has found that, with the assistance of the microscope, we can discover in the dried sand of rain-spouts a number of small bodies, which closely resemble the remains of these animalculæ, deformed through the effects of dessication. The author has taken these remains, carefully dried them on glass plates, and found that they were afterwards capable of being restored to life. On pushing his experiments further, the author found that, on applying heat at 145° or 150° Fahrenheit, the animalculæ were destroyed, and it was impossible to bring them to life again. But when they were previously dried, and all the moisture which they naturally contain was gradually expelled, they resisted a very great degree of heat; in some experiments the heat was carried to 120° C., and the animalculæ afterwards restored to life.—*Prov. Med. Journ.*, Aug. 27, 1842.

